# Altitudinal patterns and controls of plant and soil nutrient concentrations and stoichiometry in subtropical China

**DOI:** 10.1038/srep24261

**Published:** 2016-04-07

**Authors:** Xianjin He, Enqing Hou, Yang Liu, Dazhi Wen

**Affiliations:** 1 Key Laboratory of Vegetation Restoration and Management of Degraded Ecosystems, South China Botanical Garden, Chinese Academy of Sciences, Guangzhou, China; 2 University of Chinese Academy of Sciences, Beijing, China

## Abstract

Altitude is a determining factor of ecosystem properties and processes in mountains. This study investigated the changes in the concentrations of carbon (C), nitrogen (N), and phosphorus (P) and their ratios in four key ecosystem components (forest floor litter, fine roots, soil, and soil microorganisms) along an altitudinal gradient (from 50 m to 950 m a.s.l.) in subtropical China. The results showed that soil organic C and microbial biomass C concentrations increased linearly with increasing altitude. Similar trends were observed for concentrations of total soil N and microbial biomass N. In contrast, the N concentration of litter and fine roots decreased linearly with altitude. With increasing altitude, litter, fine roots, and soil C:N ratios increased linearly, while the C:N ratio of soil microbial biomass did not change significantly. Phosphorus concentration and C:P and N:P ratios of all ecosystem components generally had nonlinear relationships with altitude. Our results indicate that the altitudinal pattern of plant and soil nutrient status differs among ecosystem components and that the relative importance of P vs. N limitation for ecosystem functions and processes shifts along altitudinal gradients.

Mountains cover approximately 25% of the land surface of the Earth and host at least one-third of terrestrial plant species^1^. Mountains are useful ‘‘indicators’’ of how changes in climate may affect non-mountain terrestrial ecosystems^2^. Changes in climatic conditions (e.g., temperature and precipitation) along altitudinal gradients can influence the nutrient status^3^ and many other aspects (e.g., carbon pool) of mountain ecosystems^4^. Understanding the changes in nutrient status and nutrient dynamics along altitudinal gradients can improve our understanding about the spatial variation in nutrient cycling in mountain ecosystems and can also enhance our ability to predict how terrestrial ecosystems will respond to global warming^5^,^6^,^7^,^8^.

Changes in plant and soil nutrient concentrations along altitudinal gradients have often been studied over the last four decades^9^,^10^,^11^,^12^. In wet tropical regions, some studies have reported that foliar and litter nitrogen (N) concentrations always decrease and that foliar and litter phosphorus (P) concentrations usually decrease as altitude increases^11^,^12^, suggesting a shift from P limitation on plant growth in lowland tropical forests to N limitation in montane tropical forests^12^. These studies are generally consistent with a field fertilization study in which plant growth was found to be limited by N in high montane forests in the humid tropical Peruvian Andes but co-limited by N and P in lowland forests in Amazonia^3^. Other studies, however, have reported a lack of significant change or even an increase in foliar and/or litter N concentrations along altitudinal gradients^11^,^13^,^14^. Because low temperatures at high altitudes are likely to limit decomposition and other soil processes, researchers have proposed that soil organic carbon (C) concentration may increase while the availability of soil nutrients (e.g., N and P) may decrease with increasing elevation^11^,^15^. This view has been supported by most but not by all previous studies^11^,^16^,^17^.

Although many studies have explored the changes in plant and soil nutrient concentrations along altitudinal gradients^11^,^12^, few studies have considered this topic from a stoichiometric perspective, i.e., from a perspective that considers nutrient ratios^11^,^13^. Empirical studies have demonstrated that the vegetation N:P ratio may indicate the nature of nutrient limitation on plant growth^16^,^18^ and that the litter N:P ratio may indicate the nature of nutrient limitation on the decomposition of leaf litter^19^.

Like studies on changes in plant and soil nutrient concentrations with altitude, studies on changes in plant and soil nutrient ratios along altitudinal gradients have been inconsistent^13^,^20^. Some studies reported that leaf N:P ratios gradually decreased with increasing altitude in wet tropical regions^3^,^14^. A study of tundra ecosystems, in contrast, reported that foliar and litter N:P ratios consistently increased with altitude for heath and meadow plants at intraspecific, interspecific, and community levels^13^. Some other studies have demonstrated that altitudinal changes in nutrient ratios may differ among ecosystem components (leaves vs. mineral soil, trees vs. shrubs, etc.)^11^,^21^. The community C:N ratio in fern species, for example, declined but the soil C:N ratio increased with altitude in humid montane forests in the Bolivian Andes^21^. With an increase in altitude, the leaf N:P ratio increased for the dominant tree species but declined for the dominant shrub species in Papua New Guinea^10^. Dissimilar changes in nutrient ratios along altitudinal gradients between ecosystem components indicate the differing sensitivities of ecosystem components to environmental changes along the gradient^11^,^20^,^21^. Such dissimilar changes have important implications for the nature of nutrient limitation on different ecosystem functions (e.g., primary productivity) and processes (e.g., litter decomposition)^3^,^20^,^22^.

As shown in a recent review^11^, many ecosystem properties and processes exhibit some general patterns of change with altitude but there are also frequent exceptions. The common responses of nutrient status to changes in altitude across different regions are generally thought to be driven by changes in temperature with altitude^11^,^23^. Exceptions sometimes occur because other factors such as precipitation, parent rock, and vegetation type can vary with altitude in a different way from that of temperature^11^,^20^,^23^. Moreover, just as ecosystem processes can differ in their responses to the changes in environmental conditions with changes in altitude^11^,^24^, nutrient status may differ among ecosystem components and nutrients along altitudinal gradients^11^,^21^. Given this complexity, our capacity to predict how and why a nutrient status changes with elevation is still limited^11^,^20^,^23^. Additional research is needed to clarify how the nutrient status of key ecosystem components responds to altitude gradients, because such studies can help us understand how changes in climate may alter nutrient cycling and ultimately plant growth^11^,^15^,^23^.

This study determined the concentrations and ratios of C, N, and P in four key ecosystem components (forest floor litter, fine roots, mineral soil, and soil microbial biomass) along a 900-m altitudinal gradient (from 50 m to 950 m a.s.l.) in subtropical China. A total of 19 forest plots were assessed along this gradient. Our principal hypothesis was that these nutrient measures would change along the gradient and that the pattern of change could differ depending on the ecosystem component and the nutrient measure. Specifically, we hypothesized that (1) mineral soil C concentration would increase while plant C concentration would be constant along the altitudinal gradient; (2) the N and P concentrations in litter and fine roots would decrease with increasing altitude; and (3) the N:P ratios in litter and fine roots would decrease with altitude.

## Results

### Changes in basic site characteristics along the altitudinal gradient

**Figure 1:**
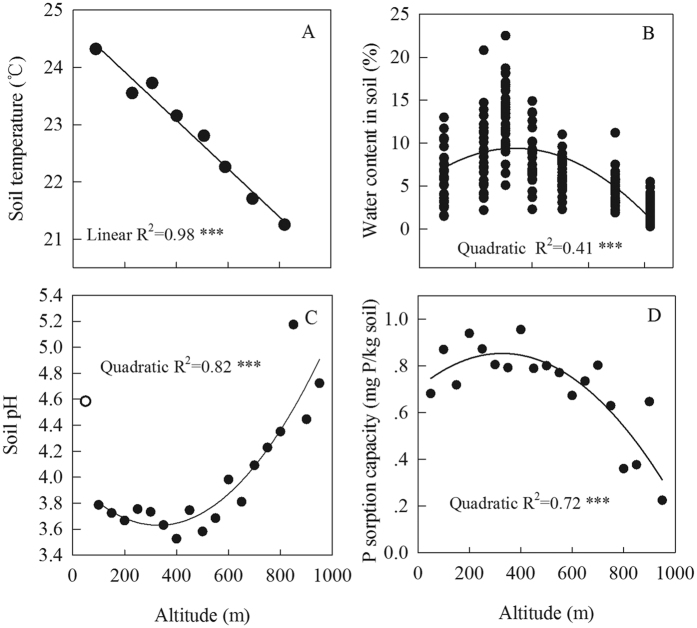
Soil temperature (**A**), moisture (**B**), pH in water (**C**), and P sorption capacity (**D**) along an altitudinal gradient in Dinghushan, South China. ^***^P < 0.001.

The average temperature of mineral soil at 0–10 cm depth decreased linearly with increasing altitude at a rate of 0.44 °C per 100 m (Fig. 1A). A similar trend was evident for soil moisture content (Fig. 1B). Soil pH tended to be constant (3.7) between 100–550 m altitude but increased linearly from a value of 4.0 at 600 m altitude to a value of 4.7 at 950 m (Fig. 1C). Soil P adsorption capacity increased from 681 mg/kg at 50 m to 954 mg/kg at 400 m and then decreased to 224 mg/kg at 950 m (Fig. 1D).

### Changes in nutrient concentrations with altitude

**Figure 2:**
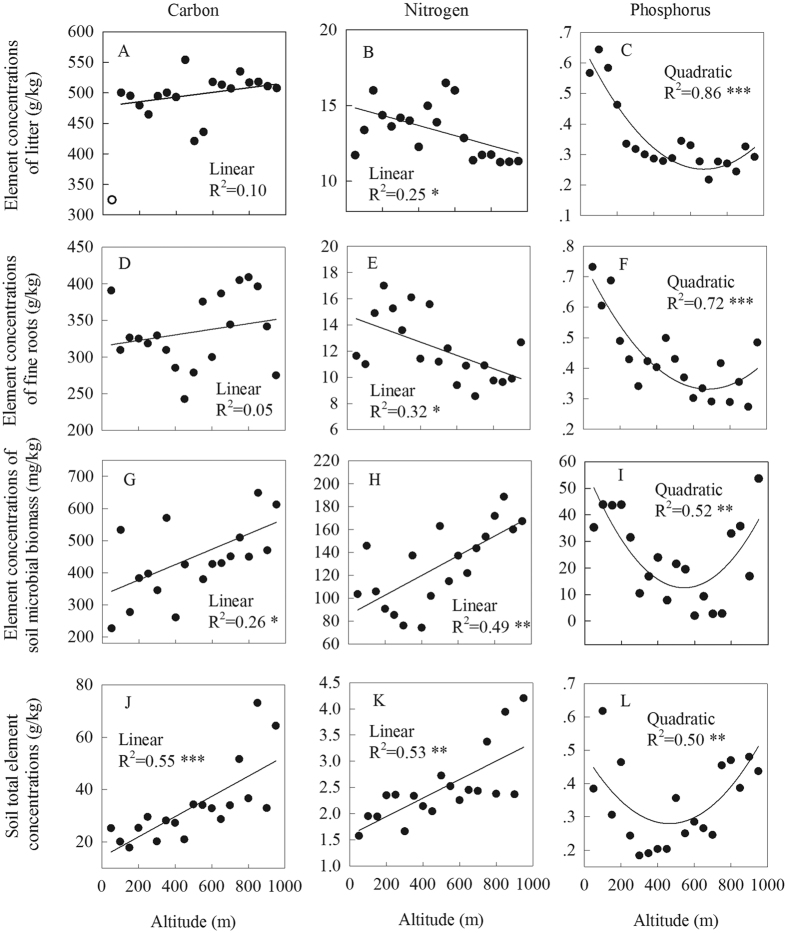
Concentrations of C, N, and P in forest floor litter, fine roots, mineral soil (0–10 cm depth) and soil microbial biomass along an altitudinal gradient in Dinghushan, South China. Litter concentration of C (**A**), N (**B**), and P (**C**); fine root concentration of C (**D**), N (**E**), and P (**F**); soil microbial biomass concentration of C (G), N (h), and P (I); soil concentration of total C (**J**), total N (**K**), and total P (**L**). ^***^P < 0.001; ^**^P < 0.01; ^*^P < 0.05. An abnormal value in (**A**) is indicted by an empty circle and was excluded from the statistical analysis.

Litter and fine root C concentrations did not significantly change with altitude (Fig. 2A,D). Soil total organic C and microbial biomass C concentrations increased linearly with altitude (Fig. 2G,J). Litter and fine root N concentrations decreased linearly with altitude (Fig. 2B,E), while the opposite was true for soil total N and microbial biomass N concentrations (Fig. 2H,K). Change in P concentration along the altitude gradient was better described by a quadratic function than by a linear one (Fig. 2C,F,I,L). The P concentrations of litter, fine roots, soil microbial biomass, and soil decreased from 50 m to 700 m altitude and then increased to 950 m altitude (Fig. 2C,F,I,L). Soil microbial biomass N was positively correlated with soil total N but negatively correlated with fine root N (Table 1). Soil microbial biomass P was positively correlated with soil total P, fine-root P, and litter P, which were positively correlated with each other.

### Changes in nutrient ratios with altitude

**Figure 3:**
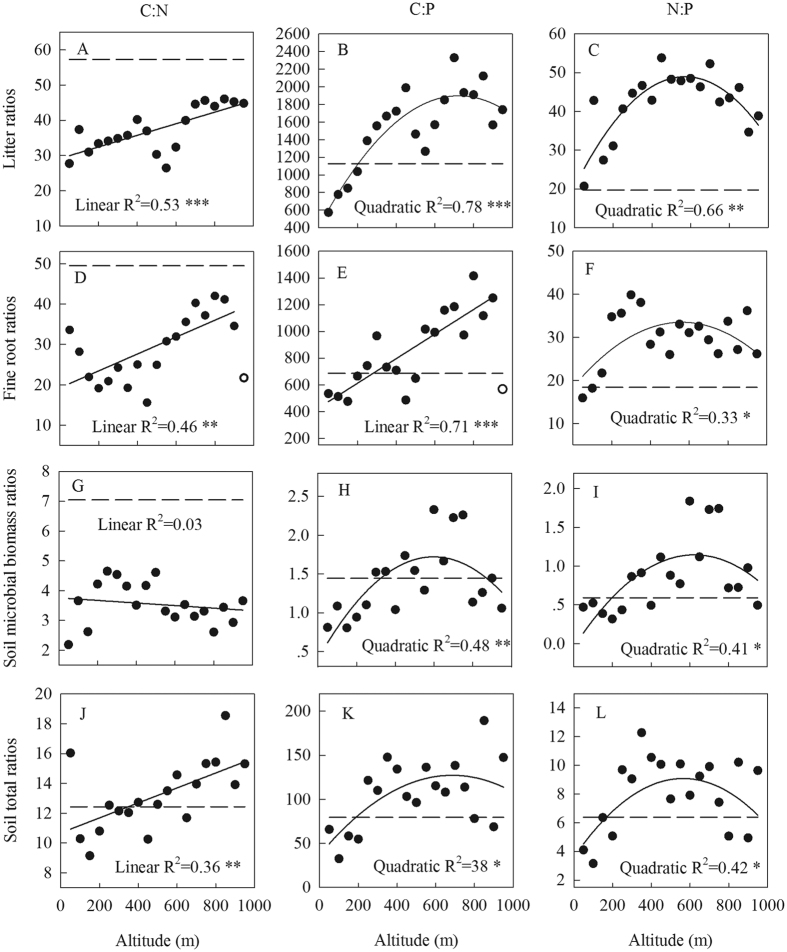
Ratios of C:N, C:P, and N:P in forest floor litter, fine roots, mineral soil (0–10 cm depth), and soil microbial biomass along an altitudinal gradient in Dinghushan, South China. Litter C:N (**A**), C:P (**B**), and N:P (**C**); fine root C:N (**D**), C:P (**E**), and N:P (**F**); soil microbial biomass C:N (**G**), C:P (**H**), and N:P (**I**); soil total C:N (**J**), C:P (**K**), and N:P (**L**). ***P < 0.001; **P < 0.01; *P < 0.05. The dashed lines represent the stoichiometric scaling between C:N:P (mass concentrations) from a global meta-analysis: forest litter averaged C:N:P ratios = 1179:20:1^56^; fine root averaged C:N:P ratios = 686:18:1^25^; forest soil microbial biomass C:N:P = 27.8:3.9:1^27^; forest soil total C:N:P = 79.4:6.4:1^27^. Soil microbial biomass C:P (**H**) and N:P (**I**) ratios were log-transformed to fit a normal distribution. Two abnormal values (one in **D** and one in **E**) are indicated as empty circles and were excluded from the statistical analysis.

While the C:N ratio of the soil microbial biomass did not change significantly with altitude (Fig. 3G), the C:N ratios of litter, fine roots, and soil all increased linearly with altitude (Fig. 3A,D,J). Plant and soil C:P and N:P ratios generally had a unimodal relationship with altitude, with highest values at the intermediate altitudes (500–700 m) (Fig. 3). Relative to global averages, the C:N ratios of litter, fine roots, and the soil microbial biomass were relatively low while most C:P and N:P ratios of fine roots, litter and soil were relatively high in the study area (Fig. 3). Soil C:N was positively correlated with fine root C:N and litter C:N, while fine root C:N was negatively correlated with microbial biomass C:N (Table 1). Litter C:P was positively correlated with soil C:P and fine root C:P, and litter N:P was positively correlated with soil microbial biomass N:P.

### Path analysis

**Figure 4:**
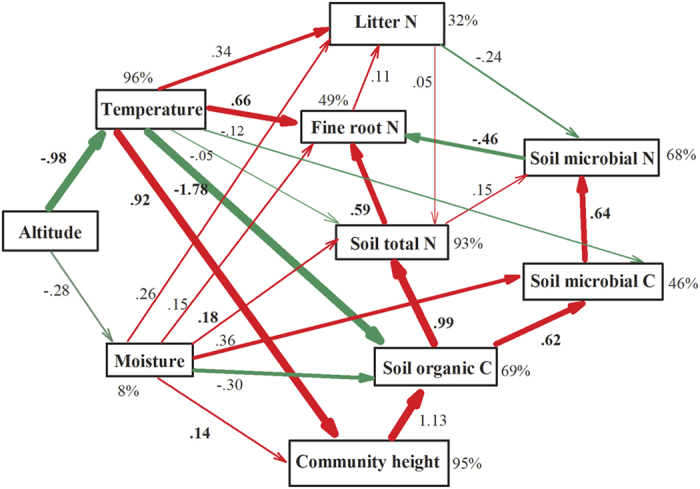
Path analysis of the changes in plant N concentration and soil C and N concentrations along an altitudinal gradient in Dinghushan, South China. Red lines indicate positive paths, and green lines indicate negative paths. Numbers on arrows are standardized path coefficients (equivalent to correlation coefficients), which are in bold if significant at *P* < 0.05. Line widths are positively related to path coefficients. Percentage near boxes indicate the variance explained by the model (R^2^).

According to the path analysis (Fig. 4), temperature had a direct and negative effect on soil organic C concentration (β = −1.78) and also an indirect and positive effect on soil organic C concentration via its influence on C input (indicated by community height) (β = 0.92 * 1.13 = 1.04); the net effect of temperature on soil organic C concentration was negative (β = −1.78 + 1.04 = −0.74). Soil microbial biomass C concentration was positively influenced by soil organic C concentration (β = 0.62). Soil total N concentration was positively influenced by soil organic C concentration (β = 0.99) and was also positively influenced by soil moisture content (β = 0.18). Soil microbial biomass N concentration was positively influenced by soil microbial biomass C concentration (β = 0.64). Fine root N concentration was positively influenced by temperature (β = 0.66), and soil total N (β = 0.59) was negatively influenced by soil microbial biomass N concentration (β = −0.46). Temperature had a direct and positive influence on litter N concentration, although the influence was not statically significant (*P* = 0.212).

## Discussion

As predicted, altitudinal changes in nutrient status differed among ecosystem components and nutrients (Fig. 2). Although plant material C concentration is typically insensitive to environmental change^25^, soil organic C concentration and microbial biomass C concentration significantly increased with altitude. Path analysis indicated that while low temperature simultaneously constrained the turnover of soil organic matter and the input of organic matter into the soil (as indicated by community height), the overall effect of temperature on soil organic C concentration was negative (Fig. 4). The path analysis also suggests that the decomposition of organic matter is more sensitive than ecosystem primary productivity to change in temperature, which has implications for the impact of global warming on C storage and fluxes in terrestrial ecosystems. In addition to the low temperature, the relatively high C:N and C:P ratios of litter, fine roots, and soil (Fig. 3) might also constrain the decomposition of litter and organic matter^26^ and therefore help explain why soil organic C concentration increased with altitude in the current study. Soil microbial activity is most commonly limited by soil C availability^27^, and therefore soil microbial biomass C, like soil organic C, also increased with altitude.

Similar as soil organic C and microbial biomass C concentrations, soil total N and microbial biomass N concentrations significantly and linearly increased with altitude (Fig 3), which could be explained by the typical close couplings between soil organic C and total N concentrations and between soil microbial biomass C and N concentrations^27^. Unlike soil total N and microbial biomass N concentrations, however, litter and fine root N concentrations linearly decreased with altitude (Fig. 2). These results are consistent with several previous studies in wet tropical regions^3^,^12^,^14^ but are inconsistent with some studies in other regions^21^,^28^. Path analysis revealed that although soil total N concentration had a positive influence on fine root N concentration, low temperature probably limited the mineralization of organic N in soil and/or the uptake of N by fine roots at high altitudes (Fig. 4). Indeed, lower rates of N mineralization at higher than at lower altitudes has been previously documented^29^. These findings are consistent with the hypothesis that low temperature slows biogeochemical cycles, including the turnover of organic matter and the uptake of nutrients by roots^30^. The direct and negative influence of soil microbial biomass N concentration on fine root N concentration (Table 1) indicates that the increases in soil microbial biomass N concentration with altitude contributed to the decreases in fine root N concentration with altitude (Fig. 2), probably as a result of the competition for N between soil microorganisms and plants^31^,^32^.

Unlike plant and soil C and N concentrations, whose changes were linearly related to altitude, the changes in plant and soil P concentrations with altitude were all better described by quadratic than by linear functions (Fig. 2). The quadratic relationship indicated a shift in the factors controlling soil total P concentration along the altitudinal gradient. The decrease in soil total P concentration from 50 m to 700 m altitude might be due to the leaching of soluble P from the upslope to the downslope^33^. For the high altitude sites (750–950 m), the increase in soil total P concentration with altitude was probably a result of the decrease in soil weathering and/or the increase in soil erosion with altitude, as suggested by the decreases in soil temperature (Fig. 1A), soil P sorption capacity (Fig. 1D), and soil depth (as observed in field) and the increase in soil pH (Fig. 1C) with altitude^34^,^35^. Because the concentrations of soil microbial biomass P and plant material P are often mainly determined by soil P availability (indicated by total P concentration)^17^,^36^ (Table 1), their changes with altitude were generally similar to that of soil total P concentration.

Plant and soil nutrient ratios can indicate the balance between nutrients in plant and soil systems and can have important implications for nutrient cycles and plant growth^37^,^38^. In this study, the C:N ratios of litter, fine roots, and soil microbial biomass (Fig. 3) were consistently lower than the global averages, and soil total C:N ratios at the low altitudes (100–350 m) were also lower than the global average, indicating the generally high N status of the studied forests and particularly of the forests at low altitudes. These results are consistent with previous studies that reported no response or even a negative response of soil microbial activity^39^ and ecosystem primary productivity^40^ to experimental N additions in low-altitude (<350 m) forests. The high N status of sites has been previously attributed to high stand age (>60 years) and to continuously high atmospheric N deposition^41^,^42^,^43^.

Significant and linear increases in the C:N ratio of litter, fine roots, and soil with altitude indicate increases in N limitation with altitude^44^ and support the hypothesis that tropical montane forests are N limited^12^. Lack of a significant change in the soil microbial biomass C:N ratio with altitude probably resulted from the strong stoichiometric homeostasis of soil microorganisms^45^. Previous studies demonstrated that heterotrophs including soil microorganisms generally have stronger C:N homeostasis than plants^17^,^46^. A recent meta-analysis of ^15^N-labelling studies revealed that soil microorganisms are generally more competitive for N than plants, especially when N supply is low^32^, which probably explains why C:N homeostasis is stronger in soil microorganisms than in plants.

Previous studies have proposed that P supply generally limits key ecosystem processes (e.g., litter decomposition) and functions (e.g., stand biomass) in the low-altitude forests of the current study area^41^,^43^. Consistent with this, the C:P and N:P ratios of the fine roots, litter, and soil samples were mostly higher than the global averages. As indicated by the nonlinear relationship between P measures and altitude, there were unimodal changes in the C:P and N:P ratios of all kinds of samples with altitude, indicating that P limitation was highest at intermediate altitudes (500–700 m). The nonlinear changes in soil P with altitude may have resulted from nonlinear changes in soil water content, pH, and P sorption capacity (Fig. 1) and from interactions among micro-climate, topography, and the vegetation; these effects on P warrant further study. The nonlinear relationships observed in this study suggest that the common assumption that plant and soil properties linearly change with altitude may not always hold^11^.

## Conclusions

This study showed that soil organic C and total N concentrations significantly increased with altitude, likely because low temperature limits the cycling of organic matter at high altitudes. Soil microbial biomass C and N concentrations exhibited similar altitudinal patterns as soil organic C and total N concentration but the opposite was true for litter and fine root N concentrations. Changes in soil total P and microbial biomass P concentrations with altitude were better described by unimodal curves than by linear functions, indicating a shift in the control of soil total P and microbial biomass P concentrations with altitude. C:N ratios of all of the studied ecosystem components significantly increased with altitude, suggesting that N may limit the decomposition of litter and soil organic matter at high altitudes. An exception was the C:N ratio of soil microbial biomass, which did not significantly change with altitude, probably as a result of the strong stoichiometric homeostasis of soil microorganisms. Unimodal distributions of plant and soil C:P and N:P ratios along the altitudinal gradient suggest that the potential for P limitation of plant growth and nutrient cycles is highest at intermediate altitudes in the study area.

## Materials and Methods

### Study sites

The study was conducted in the Dinghushan Biosphere Reserve (DHSBR), Guangdong Province, China (23°09′21″–23°11′30″N, 112°30′39″–112°33′41″E). The altitude of the Reserve ranges from 10 m to 1000 m a.s.l., with the peak located in the Reserve’s northwest margin. The Reserve has a slope ranging from 15° to 40° in most areas.

The soils are mainly lateritic red Ferralsols^47^ developed from granite. The mean annual temperature is about 21 °C and the mean annual precipitation is about 1900 mm, 70% of which occurs between May and September. The vegetation is tropical monsoon forests and subtropical monsoon evergreen broadleaf forests (Wu *et al*. 1982; He *et al*. 1982). Vegetation height declines as altitude increases (Table S1).

The basic site information for the 19 forest plots is summarized in Table S1. These 19 plots are distributed on the southeast slope of Dinghu Mountain and roughly follow the fire lane at about 50-m intervals in altitude (determined by GPS), with altitudes ranging from 50 to 950 m a.s.l. There is a classic elevation-caused vegetation change among the 19 plots. The plots are rarely impacted by humans, except the 50-m plot, which is near a tourist road.

### Sampling and analytical methods

All plots were sampled in July 2014. At each plot (20 × 30 m^2^), 10–15 sampling areas (20 × 20 cm^2^) were randomly selected. In each sampling area, apparently undecomposed forest floor leaf litter was collected, and then the underling mineral soil at 0–10 cm depth was collected using a stainless soil corer (inner diameter, 3.5 cm). Litter and fine root samples were used to represent the plant communities because they are easier to collect at the community level than fresh foliage samples. For both forest floor litter and mineral soil, samples collected from the same plot were pooled to form one composite sample. Volumetric soil samples were taken to determine soil bulk density and moisture content (g of water per 100 g of dry soil).

We collected one composite sample of each kind of sample (litter, fine roots, and mineral soil) from each of the 19 plots. This differs from the more common sampling method in which several (usually three to five) replicate samples are collected from each of a small number of (usually <10) plots. As proposed by Cottingham *et al*.^48^, the present sampling design is superior to the common one for studying the relationship between a response variable and one or more continuous independent variables (e.g., elevation) because it more is amenable to regression analysis. Regression is especially useful because it is generally more powerful than ANOVA and provides quantitative output that can be easily incorporated into ecological models.

Mineral soil samples were placed in a 4 °C refrigerator within 6 h after sampling. After the fresh weight of each sample was recorded, the soil was passed through a 2-mm sieve, and stones (>2 mm) and living fine roots (identified by the color) removed and saved. The soil was then mixed before a subsample was stored at 4 °C and subsequently used for the determination of microbial biomass C, N, and P concentrations; the remainder of the soil sample was air-dried for 2 weeks. A subsample of the air-dried soil was further ground to pass through a 0.15-mm sieve and was used for the determination of concentrations of total organic C, total N, and total P. The stones and living fine roots were washed with flowing water and then oven-dried at 65 °C for dry weight determination. The oven-dried fine roots together with oven-dried (at 65 °C) forest floor litter samples were ground to pass through a 0.15-mm sieve for the determination of concentrations of total organic C, total N, and total P.

For all plant and soil samples, total organic C and total N concentrations were determined by dry combustion with an elemental analyser (Perkin Elmer 2400 Series II); total P concentration was determined by a malachite green colorimetric method^49^ using a UV–Vis spectrometer (UV1800, Shimadzu, Japan) after digestion with nitric acid/perchloric acid.

Microbial biomass C, N, and P were determined by the chloroform fumigation extraction technique^50^: a 5-g quantity (d.w. equivalent) of soil was fumigated for 24 h at 25 °C and extracted with 0.5 M K_2_SO_4_ (for C and N) or Bray-1 (for P). Organic C and total N concentrations in the extract were determined with a Shimadzu TOC-VCPH analyser with an ASI-V auto sampler. Inorganic P (Pi) concentration in the extract was determined by a malachite green method^49^. The C, N, and P concentrations in the soil microbial biomass were calculated as the difference between the fumigated and the unfumigated samples using conversion factors of 0.45 for C^51^, 0.45 for N^52^, and 0.40 for P^53^. During extraction, some of the Pi in the solution can react with the soil colloids. This quantity of Pi was estimated by measuring the recovery of a known amount of orthophosphate P that had been added to the soil in the NaHCO_3_ extract^54^.

Phosphorus adsorption capacity was determined as the amount of P adsorbed by 1 g of dry soil following the procedure of Kovar^55^: duplicate 2.0 g air-dried soil samples were obtained, and one was added to 40 ml of 0.02 M KCl, and the other was added to 40 ml of 75 mg P/L (as KH_2_PO_4_) + 0.02 M KCl. After the samples were shaken and centrifuged, P concentrations in the supernatants were determined. The amount of P adsorbed is equal to


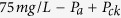


where *P*_*a*_ = P concentrations in equilibration with added P, and *P*_*ck*_ = P concentrations in equilibration without added P. The soil pH was measured at a soil/water ratio of 1:2.5.

Temperature of mineral soil at 10 cm depth was monitored hourly for 1 year with a temperature recorder (HOBO Onset U22-001, USA) in eight (elevation 100–800 m at 100-m intervals) of the 19 plots. Moisture content (g of water per 100 g of dry soil) of mineral soil at 10 cm depth was determined with a moisture probe meter (ICT International MPM-160B, Australia) three times between October and November 2014. At each time, soil moisture content was measured at 10 randomly distributed points in each plot.

### Data analysis

C:N:P ratios were calculated on a mass basis. Changes in plant and soil measures with altitude were analysed using a polynomial regression technique, with “altitude” as the independent variable and the plant or soil measure as the dependent variable. Response variables were log-transformed when necessary to satisfy assumptions of normality. Best polynomial (linear or quadratic) regressions are shown if they explained a statistically significant proportion (*P* < 0.05) of the variation. Correlations between all plant and soil measures were examined using the Pearson’s correlation method. All of these analyses were performed with SPSS 18.0 (IBM SPSS, USA).

Path analysis was performed to analyse hypothetical pathways that may explain how altitude influences the cycles of C and N in plants and soil. In our model, altitude influences the C and N concentrations of the plant and/or soil samples indirectly via its influence on the environmental conditions (temperature and moisture). Temperature can affect soil organic C concentration via its influences on both the input of organic C (represented by community height, as an indicator of community biomass and litter input) and the output of organic C (the decomposition of soil organic matter). Soil organic C concentration is the C source for soil microorganisms. Carbon concentration can also influence the N concentration in soil and in soil microorganisms because C and N concentrations are typically coupled. Nitrogen in fine roots and litter is mainly derived from the soil by sorption and transfer processes, which could be further influenced by temperature and the interaction between plant and soil microorganisms. Path analysis was performed in Amos 21.0 (IBM SPSS, USA), and the results are presented as typical path diagrams.

## Additional Information

**How to cite this article**: He, X. *et al*. Altitudinal patterns and controls of plant and soil nutrient concentrations and stoichiometry in subtropical China. *Sci. Rep.* **6**, 24261; doi: 10.1038/srep24261 (2016).

## Supplementary Material

Supplementary Information

## Figures and Tables

**Table 1:** Coefficients (*r* values) of correlations for C, N, and P concentrations and their ratios between ecosystem components^a^.

Components	C	N	P	C:N	C:P	N:P
Soil vs. SMB^b^	**0.59** a	**0.66**	**0.52**	−0.38	0.21	0.06
Soil vs. Litter	0.21	−0.36	0.42	**0.51**	**0.69**	0.42
Soil vs. FR^b^	0.29	−0.23	0.21	**0.68**	0.28	0.38
SMB vs. Litter	0.25	−0.11	**0.55**	−0.12	0.44	**0.53**
SMB vs. FR	−0.13	−**0.59**	**0.57**	−**0.62**	0.32	0.05
Litter vs. FR	−0.17	0.13	**0.81**	0.39	**0.58**	0.34

^a^Coefficients in bold are significant (*P* < 0.05).

^b^SMB: soil microbial biomass; FR: fine roots.
