# Supplementary material for: Altitudinal patterns and controls of plant and soil nutrient concentrations and stoichiometry in subtropical China
Source: Sci Rep. 2016 Apr 7;6:24261. doi: 10.1038/srep24261 (PMC4823659; doi:10.1038/srep24261)
Supplement: Supplementary Information [file srep24261-s1.pdf]

Altitudinal patterns and controls of plant and soil nutrient contents and stoichiometry in subtropical China

Xianjin He[1 2†], Enqing Hou [1†], Yang liu[1 2], Dazhi Wen [1*]

1 Key Laboratory of Vegetation Restoration and Management of Degraded Ecosystems, South China Botanical Garden, Chinese Academy of Sciences, Guangzhou, China

2 University of Chinese Academy of Sciences, Beijing, China

[*]Corresponding author; email: dzwen@scbg.ac.cn

[†] These authors contributed equally to this work.

Supplementary information

Table S1: Basic information for the 19 forest plots along an altitude gradient in the Dinghushan Biosphere Reserve, South China.

Plot number	Altitude (m)	Slope	Community height (m)	Vegetation type	Dominant species
1	45	15 °	25	Coniferous & broadleaved mixed forest	*Pinus massoniana; Eucalyptus robusta; Astanopsis fissa*
2	86	30 °	23	Ravine rainforest	*Ficus nervosa; Caryota Ochlandra; Cryptocarya chinensis.*
3	143	25 °	24		
4	201	20 °	20	Evergreen broadleaved forest	*C. chinensis; Schima superba; Cryptocarya concinna; Lindera chunii.*
5	250	32 °	19		
6	289	30 °	20	Coniferous & broadleaved mixed forest	*Schima superba; C. chinensis; P. massoniana; Craibiodendron kwangtungense; Castanopsis fissa.*
7	355	15 °	12		
8	388	30 °	15		
9	453	30 °	13		
10	499	32 °	15		
11	552	15 °	10		
12	596	25 °	6		
13	648	30 °	7	Mountainous evergreen broadleaved	*Engelhardtia chrysolepis, Bridelia monoica,*

				forest	*Machilus velutina; Rhododendron sinai.*
14	700	15 °	4		
15	750	15 °	5		
16	800	30 °	4.5	Tall shrub	*R. sinai; Corydalis pallida.*
17	849	25 °	2		
18	893	15 °	2.5	Low shrub	*Rhododendron simsii; C. pallid; Aristida chinensis; Eriachne pallescens.*
19	944	5 °	1.5		
